# Grazing exclusion is more beneficial for restoring soil organic carbon and nutrient balance than afforestation on degraded sandy land

**DOI:** 10.3389/fpls.2023.1326244

**Published:** 2023-12-21

**Authors:** Wenjie Cao, Yuqiang Li, Yun Chen, Xuyang Wang

**Affiliations:** ^1^ Northwest Institute of Eco-Environment and Resources, Chinese Academy of Sciences, Lanzhou, China; ^2^ University of Chinese Academy of Sciences, Beijing, China; ^3^ Naiman Desertification Research Station, Northwest Institute of Eco-Environment and Resources, Chinese Academy of Sciences, Tongliao, China; ^4^ Key Laboratory of Strategic Mineral Resources of the Upper Yellow River, Ministry of Natural Resources, Lanzhou, China

**Keywords:** ecological stoichiometry, ecological restoration, restoration age, nutrient limitation, soil physicochemical property

## Abstract

**Introduction:**

Vegetation restoration is an effective measure to improve the ecosystem service of degraded sandy land ecosystem. However, it is unclear how vegetation restoration on severely desertified land affect soil organic carbon (SOC) sequestration and nutrients balance. Therefore, this study was designed to clarify the response of SOC, total nitrogen (TN), total phosphorus (TP), and the resulting stoichiometric ratios (C:N:P) to afforestation and grazing exclusion, and to quantify their dynamics over time.

**Methods:**

We conducted vegetation community investigation and soil sampling in natural sparse-forest grassland (the climax community stage), afforestation (*Pinus sylvestris* var. *mongolica* (40-year, 48-year), *Caragana microphylla* (20-year, 40-year)), and grazing exclusion (20-year, 40-year) in China’s Horqin Sandy Land. Soil C:N:P stoichiometry and its driving factors under different restoration measures were then studied.

**Results:**

Afforestation and grazing exclusion significantly (*p* < 0.05) increased SOC, TN, and TP concentrations. Vegetation restoration significantly increased C:N, C:P, and N:P ratios, indicating that nutrient limitations may occur in the later stages of restoration. The C:N, C:P, and N:P ratios after a 40-year grazing exclusion were closest to those of natural sparse-forest grassland. The N:P under grazing exclusion increased from 3.1 to 4.1 with increasing restoration age (from 20 to 40 years), which was close to the national mean values (4.2). Moreover, afforestation may lead to water deficit in the surface soil. Vegetation restoration is the main factor leading to changes in soil C:N:P stoichiometry, and indirectly affects soil C:N:P stoichiometry by altering soil structure and chemical properties.

**Conclusion:**

In terms of ecological stoichiometry, grazing exclusion was more conducive to restore SOC and nutrient balance than afforestation on severely desertified land. Due to the poor soil nutrients, attentions should be paid to the soil nutrients and water conditions in the later stages of vegetation restoration. Those findings can provide valuable information for the restoration of degraded sandy land in semi-arid areas.

## 1 Introduction

Dryland covers 45% of earth’s surface and about 40% of the world’s population lives here (Li et al., 2023a). Soil degradation caused by desertification had a negative impact on soil health (e.g., loss of soil carbon (C) and nutrients, destruction of soil structure, and reduction of land productivity) (Geng et al., 2024). If degraded land continues to be exploited without any protective measures, it is highly likely to cause irreversible damage to the land productivity (Winowiecki et al., 2018). A series of ecological restoration programs around the world have been adopted (e.g., ‘Grain for Green’ project in China; the United Nations Decade on Ecosystem Restoration; the Great Green Wall in Africa) to protect vulnerable ecosystems (Han et al., 2023). However, the effect of restoration measures developed to combat desertification remains controversial.

Ecological stoichiometry studies the balance among energy and chemical elements, especially C, nitrogen (N) and phosphorus (P) in biological systems, to clarify ecological interactions and the underlying processes (Sterner and Elser, 2002). Soil C sequestration is closely related to nutrient cycling, and the fixation of C into soil organic matter (SOM) requires sufficient N and P (Chen et al., 2022a). This coupling relationship not only changes the distribution of photosynthetic products, but also regulates soil microbial metabolism and vegetation community composition (Zhang et al., 2018a; Jia et al., 2022). Stoichiometric ratios (mainly C:N:P) are crucial indicators of element cycling and functioning in ecosystems. Applying the ecological stoichiometric framework to study the biogeochemical cycling during restoration will strengthen our ability to predict the potential of soil C sequestration and manage soil fertility efficiently through additional interventions (e.g., fertilization).

Vegetation restoration is an effective measure to improve the ecosystem service of degraded ecosystem (Nadal-Romero et al., 2023). The biogeochemical cycles (mainly C, N, and P) may change during vegetation restoration, affecting plant growth, microbial community structure, and ecosystem succession processes (Deng et al., 2019; Zhang et al., 2019a). Du and Gao (2021) found that soil C and nutrients gradually increased under short-term (< 9 years) grazing exclusion in degraded alpine grassland. This may be because grazing exclusion increases soil C storage by increasing plant biomass and higher litter C:N (Du and Gao, 2021). There were some similar conclusions that soil C and N increased but P did not change significantly after the natural restoration of agricultural abandonment in the karst area of Southwest China (Lu et al., 2022). Therefore, the C:P and N:P increased, while C:N did not change significantly. The canopy density, solar radiation, soil conditions (e.g., soil moisture, pH, and soil temperature) may change during afforestation periods. This may affect soil C and nutrients by affecting the microbial activity related to organic matter decomposition (Deng et al., 2019; Zhang et al., 2019b). For example, when lands converted from farmland to grassland or plantation, soil moisture content decreased more than 35% (Yang et al., 2012). Tian et al. (2018) found that soil moisture was the primary driver explained variations of soil stoichiometry, as high soil moisture increased C and N by increasing plant biomass. Dijkstra et al. (2012) found that soil moisture is important in controlling P supply from inorganic sources. Nadal-Romero et al. (2023) found that C:N significantly increased after afforestation on abandoned farmland in the Mediterranean due to the litter input and the low decomposition rates. However, previous research mainly focused on the vegetation restoration succession after farmland abandonment and the natural restoration of degraded grasslands. We still know little about how restoration measures on severely desertified land affect soil stoichiometry.

Before 1782, the Horqin Sandy Land, located in northern China’s agro-pastoral ecotone, originally developed native vegetation dominated by grass species along with sparsely scattered woody species (Cao et al., 2023). However, due to the large-scale agricultural development and overgrazing, vegetation degradation has occurred. In addition, this region is dry and windy in the spring (Li et al., 2004). The coupling of human activities with regional climatic characteristics has led to serious aeolian desertification. Grazing exclusion and afforestation have been extensively implemented to combat desertification and restore degraded ecosystems in the Horqin Sandy Land. Effects of afforestation and grazing exclusion on C and N storage in this region has been well described (Li et al., 2012; Li et al., 2013; Li et al., 2017). However, it remained unclear that how different restoration measures affect soil stoichiometry and their dynamics over time in the Horqin Sandy Land. These knowledge gaps limit our ability to timely optimize restoration measures.

In this context, we designed the present study based on the theory of ecological stoichiometry to analyze the responses of soil organic carbon (SOC), total nitrogen (TN), and total phosphorus (TP) and the stoichiometric ratios (C:N:P) in the Horqin Sandy Land to ecological restoration. We examined *Pinus sylvestris* var. *mongolica* plantation, *Caragana microphylla* plantation, and grazing exclusion in severely degraded sandy grassland. We hypothesized that (1) soil C, N, and P would increase after afforestation and grazing exclusion on active dunes due to the improvement of environmental conditions; and (2) nutrient limitations may occur as restoration age increases. Our goals were: (i) to clarify the responses of soil C:N:P stoichiometry to afforestation and grazing exclusion over time; and (ii) to examine the relationships among vegetation factors, soil physicochemical properties, and soil C:N:P stoichiometry to identify the driving factors responsible for variations of soil stoichiometry during restoration.

## 2 Materials and methods

### 2.1 Study area

**Figure 1:**
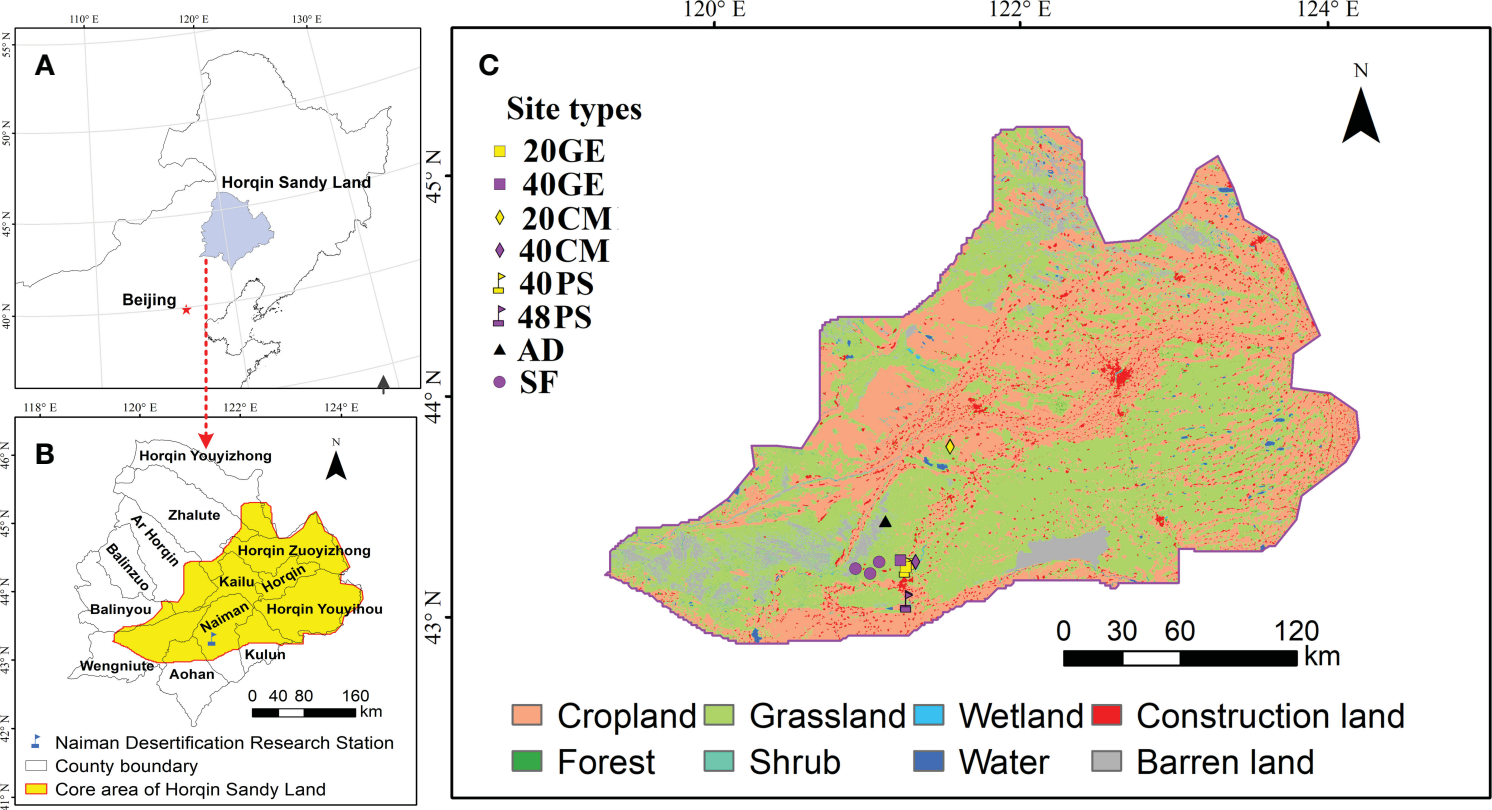
Locations of the core area of the Horqin Sandy Land and the different site types sampled in this study. Site types: 20GE, 20-year grazing exclusion; 40GE, 40-year grazing exclusion; 20CM, 20 years after planting *Caragana microphylla*; 40CM, 40 years after planting *Caragana microphylla*; 40PS, 40 years after planting *Pinus sylvestris* var. *mongolica*; 48PS, 48 years after planting *Pinus sylvestris* var. *mongolica*; AD, active dunes; SF, natural sparse-forest grassland.

The study area is in the core area of the Horqin Sandy Land, which is a Chinese national ecological function zone for preventing and mitigating aeolian desertification (MEE and CAS, 2015). The study area (with a temperate semi-arid continental climate) is located east of China’s Inner Mongolia (42.71°N to 44.83°N and 118.86°E to 123.70°E) (**Figure 1**). The mean annual temperature ranges from 3 to 7°C. The mean annual precipitation ranges from 350 to 500 mm, of which 70% falls from July to September. The annual average potential evaporation ranges from 1500 to 2500 mm. The annual average wind speed ranges from 3.4 to 4.4 m·s^−1^ (Duan et al., 2014). The annual sandstorm frequency is 10 to 15 days and the storms mainly occur in the spring.

The zonal soils are Kastanozems and Chernozems (FAO, 2006) derived from Quaternary alluvial sediments, but the soils have changed to Cambic Arenosols in large areas due to desertification. The native vegetation is dominated by palatable grass species with sparsely scattered woody species, which are dominated by *Ulmus macrocarpa*, *Ulmus pumila*, *Crataegus pinnatifida*, *Armeniaca (Prunus) sibirica*, and *Lespedeza bicolor* (Liu and Zhao, 1993). The vegetation has been dominated by xerophytes and psammophytes as a result of excessive grazing and agricultural reclamation, with the dominant species being *Salix gordejevii*, *Periploca sepium*, *C. microphylla*, *Artemisia halodendron*, and *Pennisetum flaccidum* (Liu and Zhao, 1993).

### 2.2 Site selection

We conducted field investigation and sampling from July to August 2022 using the space-for-time substitution approach. Our study was performed near the Naiman Desertification Research Station of the Chinese Academy of Sciences (42.93°N, 120.70°E, 377 m asl.). Based on site investigations and site location information obtained in our previous research (Li et al., 2012; Li et al., 2013; Li et al., 2017), we selected the following restoration site types: (1) 38-year-old and 48-year-old *P. sylvestris* plantation, (2) 20-year-old and 40-year-old *C. microphylla* plantation, and (3) 22-year-old and 41-year-old grazing exclusion sites (**Figure S1**). Before implementing restoration, all of the site types were active dunes, which had similar soil parent material, climate, and topography and not been subjected to any management measures. For the convenience of statistical analysis, we defined 22-year-old and 41-year-old grazing exclusion sites as 20-year-old and 40-year-old grazing exclusion sites, respectively; and defined 38-year-old *P. sylvestris* site as 40-year-old *P. sylvestris* site. In addition, we surveyed areas with active dunes (i.e., severely degraded sandy grassland, with an equivalent restoration age of 0 years) and natural sparse-forest grassland (the climax community stage) as references (**Figure S1**). In total, we examined eight site types (**Figure 1**, **Table S1**), and each site type comprised three independent replicates. We randomly established three 20 m×20 m plots at each site for trees and shrubs investigations (**Table S2**), and established three 1 m×1 m quadrats along the diagonal of each plot at an interval of 10 m for herbs investigation and soil sampling.

### 2.3 Vegetation community investigation and soil sampling

The species composition, the density, height, and vegetation cover of each herbaceous species were recorded in each quadrat. The aboveground living herbaceous plants and litter were then collected. We performed soil sampling in the same quadrats where the vegetation had been removed. For the grazing exclusion, natural sparse-forest grassland, and active dune sites, we collected five soil samples to a depth of 20 cm from the quadrats using a soil auger with a diameter of 2.5 cm (**Figure S2A**) and then combined them to produce a well-mixed composite sample. For the afforestation sites, we tried to avoid the “islands of fertility effect” of shrubs and the possible effect of tree roots on the rhizosphere soil. We then randomly established three sampling locations (corresponding to the three 1 m × 1 m quadrats) along the diagonal of the surrounding plot at an interval of 10 m in each plot. We collected soil samples at 20-cm intervals between woody plants adjacent to the vegetation sampling location (**Figure S2B**) and then mixed them thoroughly to form a composite sample. The soil samples were sieved through a 2-mm mesh to remove stones, animal debris, and plant residues, then were transported to the laboratory. In total, we collected 216 soil samples (eight site types × three replicate sites × three replicate plots × three replicate quadrats). We chose two additional locations in each quadrat to collect soil cores to a depth of 20 cm using a cylindrical 100-cm^3^ sampler to determine soil bulk density (BD), field water capacity (FC), and the saturated water content (SAW).

### 2.4 Soil property and plant biomass measurements

Soil samples were air-dried before analysis of the physicochemical properties. We determined SOC, TN, and TP concentrations (in g·kg^−1^) using the Walkley-Black dichromate oxidation method, Kjeldahl method, and molybdenum–antimony colorimetric method, respectively (Chen et al., 2022c). Soil pH (1:2.5 w:v) and electrical conductivity (EC, in μS·cm^−1^; 1:5 w:v) were measured in deionized water using PHS-3C and DDS-307A instruments (INESA Scientific Instrument Co., Ltd., Shanghai, China), respectively. Soil water content (SWC) and BD were measured after oven-drying the soil samples at 105 °C for 48 hours to constant weight. The living plants and litter were dried at 75 °C for 48 hours to constant weight and then weighed to measure the aboveground living biomass (AGB, in g·m^−2^) and litter biomass (LB, in g·m^−2^), respectively. We determined FC, SAW, and the particle-size distribution using the methods described by Chen et al. (2022b). The soil samples were separated into five fractions: coarse sand (from 2.00 to 0.50 mm), medium sand (from 0.50 to 0.25 mm), fine sand (from 0.25 to 0.10 mm), very fine sand (from 0.10 to 0.05 mm), and silt and clay (<0.05 mm). We also calculated the Shannon–Wiener diversity index (*H*) in each quadrat (Ma and Liu, 1994).


$$H=-\sum_{i=1}^{S}P_{i}\text{ln}P_{i}$$

Where *S* represents the total number of species in a quadrat, *P_i_* is the proportion of the total number of species accounted for by a given species “*i*”.

### 2.5 Statistical analyses

We conducted normality and homogeneity of variance tests using the Kolmogorov-Smirnov test and the Levene test, respectively. If needed, we used ln-transformation to meet the assumptions of normality and homogeneity. Two-way ANOVA was used to analyze the effects of restoration measures, restoration ages, and their interactions on soil C:N:P stoichiometry. We then used one-way ANOVA followed by LSD test to test for differences in soil C:N:P stoichiometry among different restoration measures and restoration ages at the significant level of α = 0.05. The statistical analyses were conducted in SPSS (version 20.0; https://www.ibm.com/analytics/spss-statistics-software). We used Origin 2018 (https://www.originlab.com/) to perform linear regression to fit the relationships among SOC, TN, and TP. We calculated Pearson’s correlation coefficient (*r*) using the “cor” function in the R software to quantify the relationships among soil C:N:P stoichiometry and environmental factors. We used version 0.92 of the “corrplot” package for R (https://cran.r-project.org/web/packages/corrplot/index.html) to visualize the correlation matrix. Given the strong correlation between factors, we operated principal component analysis (PCA) (He et al., 2022) using SPSS to achieve dimensionality reduction and calculate scores (**Table S3**). Structural equation modeling (SEM) was then used to quantify the relative contribution of the factors regulating soil C:N:P stoichiometry using AMOS 24.0 (http://amosdevelopment.com/). The standardized path coefficients were used to indicate correlations between factors in SEM.

## 3 Results

### 3.1 Soil C:N:P stoichiometry under different restoration measures and ages

**Figure 2:**
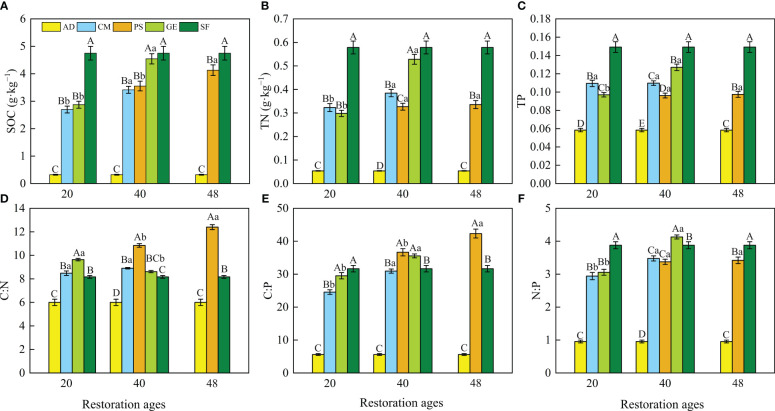
**(A)** Soil organic carbon (SOC), **(B)** total nitrogen (TN), and **(C)** total phosphorus (TP), and the **(D)** soil organic carbon:total nitrogen (C:N) ratio, **(E)** soil organic carbon:total phosphorus (C:P) ratio, and **(F)** total nitrogen:total phosphorus (N:P) ratio for the eight site types. Different lowercase letters indicate significant difference among different restoration ages at the same site types (*p* < 0.05). Different capital letters indicate the significant difference among different site types at the same restoration age (*p* < 0.05). Values are means ± SE (n=27 for each site type). AD, active dunes; SF, natural sparse-forest grassland; CM, *C. microphylla* plantation; GE, grazing exclusion; PS, *P. sylvestris* plantation.

The restoration measures and restoration ages significantly affected SOC, TN, and TP and the resulting stoichiometric ratios (**Figure 2** and **Table 1**). Two-way ANOVA indicated that the SOC, TN, TP, C:N, and N:P were significantly affected by both site types and restoration ages and their interaction (**Table 1**; *p *< 0.01). The C:P was affected by site types and restoration ages independently (*p* < 0.01), while C:P was not affected by the interaction between site types and restoration ages (*p* > 0.05). The SOC, TN, and TP concentrations were the highest in the natural sparse-forest grassland. The SOC, TN, TP concentrations, and C:N, C:P, and N:P ratios of soils under the three restoration measures were significantly (*p* < 0.05) greater than those in the active dunes, with increases of 7.4 to 13.1, 4.5 to 8.8, 0.6 to 1.2, 1.4 to 2.1, 4.4 to 7.6, and 3.1 to 4.3 times, respectively. The 40-year grazing exclusion had the highest SOC, TN, and TP among the restoration measures. In addition, the SOC, TN, and TP concentrations and three stoichiometric ratios of the 40-year grazing exclusion were closest to the corresponding values of natural sparse-forest grassland. The SOC, TN, TP, C:P, and N:P of the 40-year grazing exclusion increased by 58.2, 77.1, 30.9, 20.5, and 35.1%, respectively, compared with the 20-year grazing exclusion. In contrast, the C:N of the 40-year grazing exclusion decreased by 10.6% compared with the 20-year grazing exclusion. Compared with the 20-year *C. microphylla* plantations, the SOC, TN, C:P, and N:P of the 40-year *C. microphylla* plantations increased by 26.6, 19.0, 26.0, and 18.3%, respectively. The TP and C:N of *C. microphylla* plantations did not change significantly with increasing restoration age (*p* > 0.05). Compared with the 40-year *P. sylvestris* plantations, the SOC, C:N, and C:P of the 48-year *P. sylvestris* plantations increased by 16.3, 14.4, and 15.5%, respectively. The TN, TP, and N:P of *P. sylvestris* plantations did not change significantly (*p* > 0.05) with increasing restoration age. In addition, the SWC content of *C. microphylla* and *P. sylvestris* plantations decreased with the increase of afforestation years.

**Table 1:** The two-way ANOVA on the effects of site types and restoration ages on soil organic carbon (SOC), total nitrogen (TN), and total phosphorus (TP) concentrations and the resulting stoichiometric ratios (C:N:P).

Parameters	Site types	Restoration ages	Site types × Restoration ages
SOC	10.5 (<0.001)	30.6 (<0.001)	8.7 (<0.01)
TN	26.0 (<0.001)	37.6 (<0.001)	25.1 (<0.001)
TP	17.8 (<0.001)	12.2 (<0.001)	23.5 (<0.001)
C:N	68.7 (<0.001)	29.1 (<0.001)	23.4 (<0.001)
C:P	17.1 (<0.001)	30.4 (<0.001)	0.0 (0.9) ^ns^
N:P	17.0 (<0.001)	40.9 (<0.001)	9.0 (<0.01)

The values in the table are F statistic and probability level, respectively; ns, not significant.

### 3.2 The relationships among SOC, TN, and TP under different restoration measures

Except for active dunes, we found a significant positive linear relationship between SOC, TN, and TP for all site types (*p* < 0.001, **Table 2**, **Figure S3**). The *R* ^2^ values for the C–N, C–P, and N–P relationships of the other seven site types were 0.85 to 0.98, 0.39 to 0.86, and 0.47 to 0.91, respectively (**Table 2**). As restoration age increased, the *R* ^2^ values for the C–P and N–P relationships increased under grazing exclusion, whereas the *R* ^2^ for *C. microphylla* plantations increased for all three relationships. In contrast, *R* ^2^ of the C–N and C–P relationships for *P. sylvestris* plantations decreased, and *R* ^2^ of N–P in *P. sylvestris* plantations remained relatively constant with increasing restoration age (**Table 2**).

**Table 2:** The relationships among soil organic carbon (SOC), total nitrogen (TN), and total phosphorus (TP) for the eight site types.

Relationship	Site type	Fitted equations	*R* ^2^	Pearson’s *r*
Between SOC and TN (C–N)	SF	*y*=0.11*x*+0.08	0.92	0.96***
	AD	*y*=0.02*x*+0.05	0.01	0.21
	20GE	*y*=0.10*x*+0.01	0.94	0.97***
	40GE	*y*=0.11*x*+0.03	0.93	0.97***
	20CM	*y*=0.13*x*−0.02	0.85	0.92***
	40CM	*y*=0.12*x*−0.02	0.98	0.99***
	40PS	*y*=0.08*x*+0.04	0.93	0.96***
	48PS	*y*=0.08*x*−0.01	0.88	0.94***
Between SOC and TP (C–P)	SF	*y*=0.02*x*+0.06	0.66	0.82***
	AD	*y*=0.02*x*+0.05	0.03	0.18
	20GE	*y*=0.01*x*+0.06	0.39	0.65***
	40GE	*y*=0.02*x*+0.05	0.86	0.93***
	20CM	*y*=0.02*x*+0.05	0.63	0.80***
	40CM	*y*=0.02*x*+0.05	0.72	0.85***
	40PS	*y*=0.01*x*+0.05	0.61	0.79***
	48PS	*y*=0.01*x*+0.05	0.52	0.74***
Between TN and TP (N–P)	SF	*y*=0.17*x*+0.05	0.64	0.81***
	AD	*y*=−0.20*x*+0.07	0.02	−0.14
	20GE	*y*=0.13*x*+0.06	0.48	0.71***
	40GE	*y*=0.16*x*+0.04	0.91	0.96***
	20CM	*y*=0.14*x*+0.06	0.47	0.70***
	40CM	*y*=0.14*x*+0.06	0.73	0.86***
	40PS	*y*=0.15*x*+0.05	0.69	0.84***
	48PS	*y*=0.16*x*+0.04	0.71	0.85***

*** p < 0.001. SF, natural sparse-forest grassland; AD, active dunes; 20GE, 20-year grazing exclusion; 40GE, 40-year grazing exclusion; 20CM, 20-year C. microphylla plantation; 40CM, 40-year C. microphylla plantation; 40PS, 40-year P. sylvestris plantation; 48PS, 48-year P. sylvestris plantation.

### 3.3 Factors controlling variation of soil C:N:P stoichiometry

**Figure 3:**
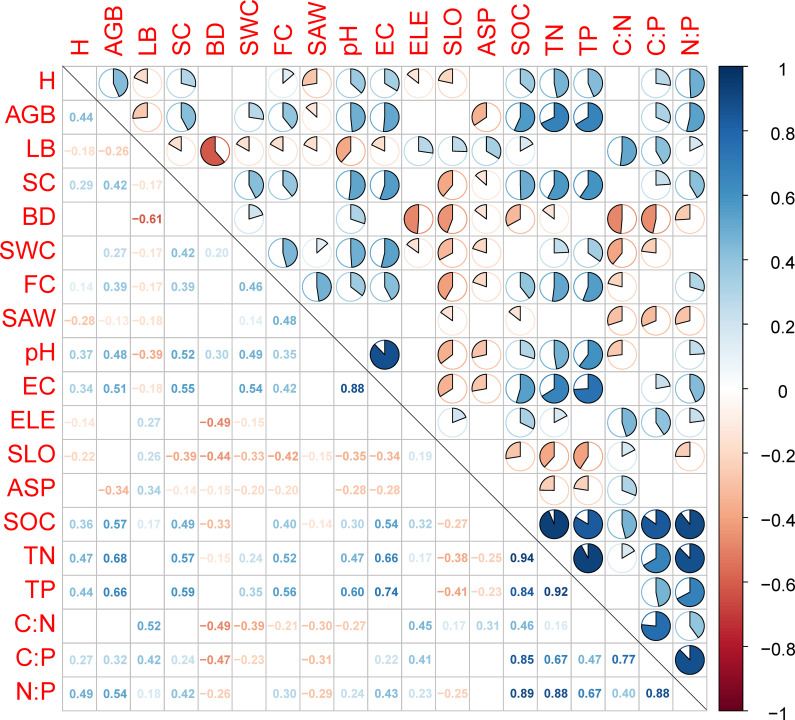
The correlation coefficient matrix (Pearson’s *r*) for the relationships between pairs of environmental factors and soil organic carbon (SOC), total nitrogen (TN), and total phosphorus (TP) and the resulting stoichiometric ratios (C:N:P). Numbers below the main diagonal are the correlation coefficients (*p* < 0.05). Above the main diagonal, correlation coefficients are displayed in a pie chart. A positive correlation is represented by blue (*p* < 0.05), whereas a negative correlation is displayed as red (*p* < 0.05). Cells with a non-significant correlation coefficient are not shown (*p* > 0.05). Variables: AGB, aboveground living biomass; ASP, aspect; BD, soil bulk density; C:N, soil organic carbon:total nitrogen; C:P, soil organic carbon:total phosphorus; EC, electrical conductivity; ELE, elevation; FC, field water capacity; H, Shannon–Wiener diversity index; LB, litter biomass; N:P, total nitrogen:total phosphorus; SAW, saturated water content; SC, silt and clay; SLO, slope; SWC, soil water content.

**Figure 4:**
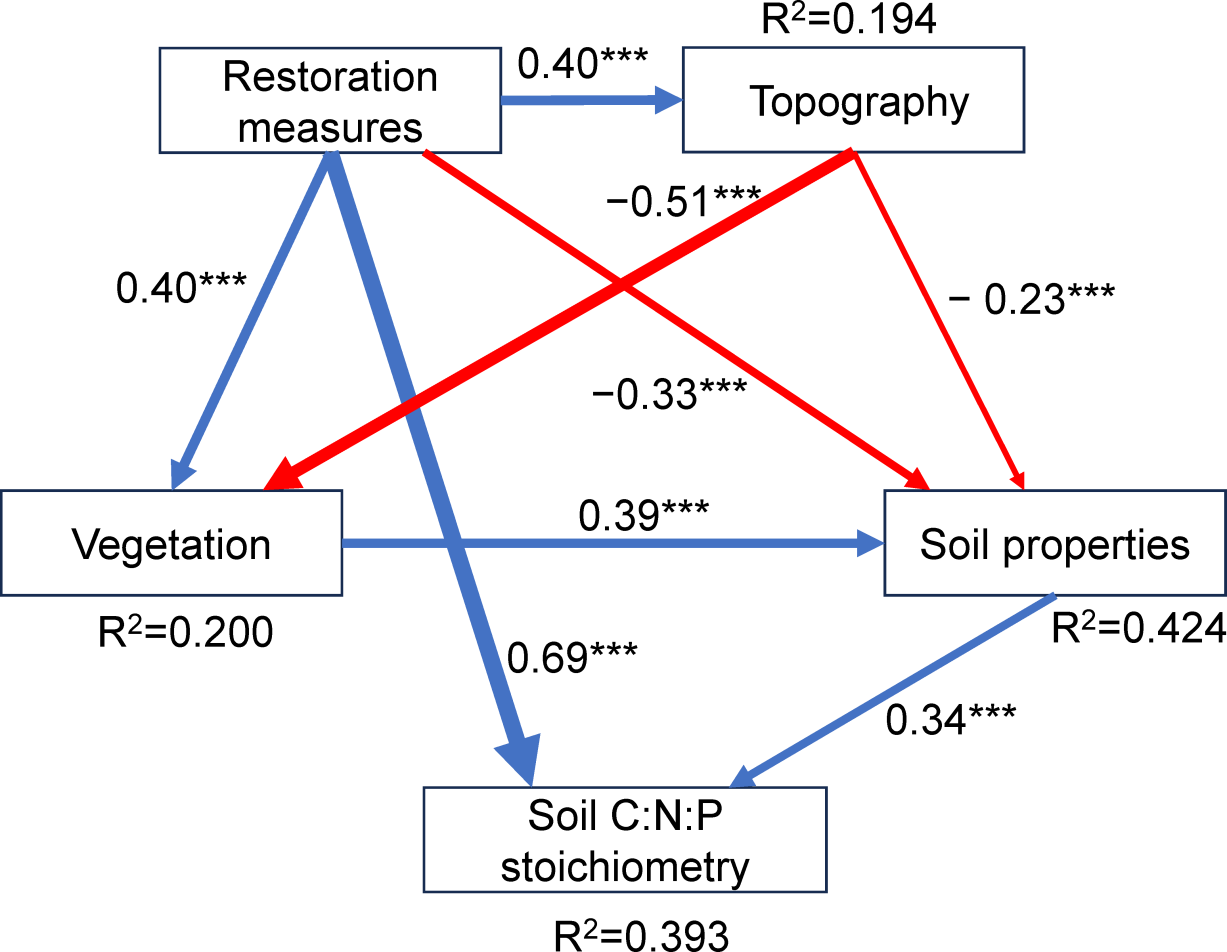
Structural equation models (SEM) revealing the direct and indirect effects of environmental factors and restoration measures on soil C:N:P stoichiometry. The numbers are the standardized path coefficients and *R* ^2^. Blue and red solid arrows indicate positive and negative associations, respectively. Non-significant paths are not shown. ***p < 0.001. The model fit summary: *p* = 0.59, χ^2^/df = 0.53, GFI = 1.00, AGFI = 0.99, CFI = 1.00, RMSEA = 0.00.

Silt and clay contents, EC, *H*, and AGB were positively correlated with SOC, TN, TP, C:P and N:P. Elevation and LB were positively correlated with SOC, C:N, C:P and N:P. Soil BD and SAW were negatively correlated with SOC, C:N, C:P and N:P. Soil pH, SWC, and FC were positively correlated with TN and TP, but negatively correlated with C:N. Slope and aspect were negatively correlated with TN and TP, but positively correlated with C:N (*p* < 0.05, **Figure 3**). Soil C:N:P stoichiometry was directly affected by restoration measures and soil properties (**Figure 4**). Restoration measures exhibited the highest direct effect (with the standardized path coefficient of 0.69, *p* < 0.001) on soil C:N:P stoichiometry (**Figure 4**). In addition, restoration measures and topography indirectly affected soil C:N:P stoichiometry by regulating vegetation and soil properties. Vegetation indirectly affected soil C:N:P stoichiometry by regulating soil properties (**Figure 4**). All predictor variables together explained 39.3% of the variation in soil C:N:P stoichiometry.

## 4 Discussion

### 4.1 Changes in C:N:P stoichiometry with vegetation restoration

Afforestation and grazing exclusion in areas with active dunes can help to reverse desertification by enhancing soil fertility. Overall, soil C:N:P stoichiometry of the 40-year grazing exclusion were closest to those of the natural sparse-forest grassland, indicating that the restoration effect of grazing exclusion was better than afforestation in the severely degraded sandy grassland under the perspective of soil C:N:P stoichiometry. The result was consistent with previous study which found that the soil health index increased after 21-year-old grazing exclusion in Brazilian drylands and was close to the level of native vegetation (Lima et al., 2024). However, the SOC, TN, and TP concentrations in the eight site types were significantly lower than previously reported values in Chinese (24.6, 1.9, and 0.8 g·kg^−1^, respectively; Tian et al., 2010) and in global terrestrial ecosystems (57.2, 4.1, and 0.5 g·kg^−1^, respectively; Xu et al., 2013). In addition, the SOC, TN, and TP concentrations of grazing exclusion and *P. sylvestris* sites in our research were lower than those in the fenced and afforested soils of China’s Loess Plateau, respectively (Zeng et al., 2016; Bai et al., 2019), which had a shorter restoration time. These results indicated that SOC and nutrients in the Horqin Sandy Land were very low. Although vegetation restoration increases the input of plant sources, it has a lower decomposition rate in desertification areas (Zechmeister-Boltenstern et al., 2015). Moreover, compared to vegetation restoration, the restoration of soil fertility is more difficult, and usually takes decades or even hundreds of years in the severely degraded sandy grasslands (Li et al., 2007).

Except for the 48-year *P. sylvestris*, whose C:N (12.4) was slightly higher than that in China as a whole, the C:N values of the remaining seven site types and the C:P and N:P values of all site types in the Horqin Sandy Land were lower than those in China as a whole, with values of 12.4, 52.7, and 4.2, respectively (Tian et al., 2010) and in global terrestrial ecosystems, with values of 14.1, 111.1, and 7.9, respectively (Xu et al., 2013). The C:N of the 20-year grazing exclusion in our research was higher and C:P and N:P were lower than those in the 17 years of grazing exclusion in degraded semi-arid region in Brazil (Filho et al., 2019). Moreover, the C:N of the 48-year *P. sylvestris* plantations in our research was higher than that (10.3) in the 50-year afforested soils of Mediterranean mountain agroecosystems (Nadal-Romero et al., 2023). These results indicated that soil in our study region lacks C and N. In other words, the decomposition rate of plant litter is relatively slow, resulting in insufficient soil C and N accumulation (Zechmeister-Boltenstern et al., 2015). Wang et al. (2014) suggested that the C:N ratio was relatively consistent under different land-use and cover types in a subtropical wetland, including sites that received nitrogenous fertilizer. There is a strong positive correlation between C and N (*r* = 0.94) in our study. Because N is distributed in several types of organic polymers and humus, and organic N is both a N source and a C source. Therefore, when microorganisms decompose organic N, they also affect the mineralization flux of C (Zechmeister-Boltenstern et al., 2015). As a result, the synchronous changes of C and N lead to relatively stable ratios. The tight coupling of C and N for most site types supports this conclusion.

With increasing restoration age, the N:P under grazing exclusion increased from 3.1 to 4.1, which was close to the national mean values (4.2) (Tian et al., 2010) and much higher than that in active dunes (1.0). In addition, the enhanced N–P relationship under grazing exclusion supports the conclusion that soil N:P converged after long-term grazing exclusion in Inner Mongolia grasslands (Yang et al., 2017). The C:P and N:P of 20-year *C. microphylla* plantations were higher than those of a 20-year-old *Caragana korshinskii* plantation on the Loess Plateau (Chen et al., 2022a). The N:P of *C. microphylla* plantations increased with increasing age, suggesting that N limitation was mitigated but P limitation was exacerbated with increasing time since restoration, which partially supports our second hypothesis. Phosphate fertilizers should therefore be applied at *C. microphylla* sites in the later stage of the restoration. There may be two reasons for these changes: First, the silt and clay content at *C. microphylla* sites increased significantly compared with that in active dunes because *C. microphylla* has pinnate leaves that can capture blowing soil fine particles (which are rich in C and N) and plant debris (Zhao et al., 2007). Second, *C. microphylla* is a leguminous plant species that can fix N (Li et al., 2023b).

*Pinus sylvestris* var. *mongolica* afforestation greatly improved soil fertility, because afforestation both stabilizes dunes by reducing wind speed and contributes a large amount of high-quality litter to the soil (Zhang et al., 2019b; Wu et al., 2020). However, *P. sylvestris* is a tree species with a shallow root system, resulting in a vulnerability to fluctuations in soil moisture and severe drought stress in the shallow rooting zone (Song et al., 2017). Previous studies have shown that vegetation restoration decreased SWC in arid and semi-arid areas (Deng et al., 2019), and the low moisture content may have decreased microbial biomass and decomposition rate of litter (Borken et al., 2006). In addition, soil N mineralization was affected by changes in soil moisture (Beier et al., 2008). In this study, soil moisture decreases with increasing age of *P. sylvestris* afforestation may result in insignificant changes in N and P nutrients and increasing C:N with increasing age (Beier et al., 2008; Tao et al., 2020). Soil in these plantations may become N-limited over time. We therefore recommend measures such as irrigation and application of nitrogenous fertilizer in the later stages of *P. sylvestris* afforestation to avoid the development of dried soil layers and to improve soil fertility.

### 4.2 Effect of environmental factors on soil C:N:P stoichiometry

Previous studies have shown that soil stoichiometry in restored ecosystems was most strongly affected by the vegetation type (Su et al., 2019; Wang et al., 2022), restoration age (Su and Shangguan, 2021), soil properties (Yang and Liu, 2019), and management measures (Tao et al., 2021). In the present study, vegetation restoration is the main reason for improving SOC sequestration and soil fertility in severely desertified land. Those improvements enhanced markedly over time after vegetation restoration, which is consistent with the previous study of shrub afforestation in the Tengger Desert (Yang et al., 2014). These results supported our first hypothesis. Firstly, afforestation and grazing exclusion have formed actual protected areas in the areas with active dunes, increasing surface roughness to reduce wind speed and reducing human activities interference (e.g., grazing), which protect soil from wind erosion (Han et al., 2023). Secondly, afforestation and grazing exclusion have increased the productivity, species richness, and diversity, resulting in the increase of soil C and nutrient sources by increasing the quantity and quality of litter and root exudates (Lange et al., 2015; Yang et al., 2019; Zhang et al., 2019b). In addition, the diversity of soil root exudates and litter inputs after restoration not only stimulates microbial metabolism, but also provides abundant energy and nutrients for decomposers, which is beneficial for the accumulation of C, N, and P (Zhang et al., 2018b). The previous study found that roots had a greater impact on soil nutrients than other plant components, which was related to the rhizosphere microbial community and root exudates (Zeng et al., 2016). Although we did not attempt to quantify the contributions of roots to SOM and nutrients, the sampling depth was only 20 cm in the present study, which was the main rooting zone of herbaceous plants (Yang et al., 2017). The increase in herbaceous plant richness promotes the improvement of fertility in the topsoil due to the short life history, the large amount of root residues, and high litter turnover rate of herbaceous plant (Wei et al., 2012; Wang et al., 2021). This may partially explain why the effectiveness of the grazing exclusion is better than afforestation.

Soil properties are another important influencing factor for soil C:N:P stoichiometry, which is consistent with previous study (Tian et al., 2018). Vegetation restoration and changes in topography caused by grassland degradation–restoration process affects soil C:N:P stoichiometry by altering soil structure and chemical properties. Soil texture and BD are important parameters for evaluating soil compaction strength and soil structure, and play an important role in regulating the litter decomposition, root growth, species composition, and community structure (Dodd et al., 2002; Don et al., 2011). Long term vegetation restoration reduces BD and increases soil fine particulate matter, promoting the formation of soil aggregates. In this context, it is beneficial for SOC sequestration and nutrient accumulation (Zhou et al., 2008; Chen et al., 2022b; Wang et al., 2022). Soil BD is greatly affected by porosity, and soils with low BD were porous. Therefore, soil texture and structure typically alter soil moisture and have an impact on soil C:N:P stoichiometry. Because SWC can change litter decomposition and nutrient release mediated by decomposers (Cui et al., 2020). Borken et al. (2006) showed that low SWC adversely affected microbial metabolism. Water is the main limiting factor for plant primary productivity in arid and semi-arid regions (Wang et al., 2018), and this may explain the negative correlation between SWC and the C:N ratios and the positive correlation between SWC and nutrients. Therefore, soil water deficit caused by the increase in afforestation years may have adverse effects on SOC sequestration and soil fertility.

Soil pH and EC are parameters of soil quality, and have an impact on soil C:N:P stoichiometry by affecting soil enzyme and microbial activity (Zhang et al., 2019c; Qiao et al., 2020; Zhang et al., 2021). Soil pH and EC also indirectly change the microbial community by changing the soil structure and the availability of nutrients (Chen et al., 2022b). For example, bacterial and fungal communities differed significantly between soils with different pH (Puissant et al., 2019). Fungi have lower nutrient requirements and slower metabolic activity than bacteria, so they affect soil stoichiometry at different rates (Zechmeister-Boltenstern et al., 2015; Zhou et al., 2023). An experiment with manipulation of desert grasslands in northern China showed that the availability of nitrate N increased after soil acidification, whereas soil alkalization reduced total C and TN (Liu et al., 2022). In the present study, we found a positive correlation between pH and TP, which was inconsistent with the conclusions drawn by Wang et al. (2014) in subtropical wetlands. This may be due to the large climatic differences between the two regions.

## 5 Conclusion

Our results indicated that afforestation and grazing exclusion in active dunes contributed to increasing SOC, TN, and TP. The SOC, TN, and TP and the resulting stoichiometric ratios in the 40-year grazing exclusion were close to those of natural sparse-forest grassland (which represents the undamaged natural ecological condition), indicating that grazing exclusion was more beneficial for restoring SOC and nutrient balance than afforestation. With increasing duration (from 20 to 40 years), the N:P increased under grazing exclusion, which was close to the national mean values. In addition, the *R* ^2^ of the N–P relationship under grazing exclusion increased, indicating that N:P converged with increasing time since restoration. The N:P of *C. microphylla* sites increased with increasing restoration age (from 20 to 40 years), suggesting that N limitation was mitigated whereas P limitation was exacerbated. Therefore, phosphate fertilizers should be applied later during the restoration process. With increasing afforestation age (from 40 to 48 years), the SOC and C:N in the *P. sylvestris* plantation increased. Therefore, the soil may be subject to an increasing N limitation over time. We recommend applying nitrogenous fertilizers in the later stages of *P. sylvestris* afforestation. Moreover, soil drought stress may be a challenge that plantations will face in the future. Vegetation restoration is the main factor directly leading to changes in soil C:N:P stoichiometry, and indirectly affects soil C:N:P stoichiometry by altering soil structure and chemical properties. Our findings provide a framework for linking ecological restoration measures to soil stoichiometry in semi-arid regions.

## Data availability statement

The raw data supporting the conclusions of this article will be made available by the authors, without undue reservation.

## Author contributions

WC: Investigation, Software, Visualization, Writing – original draft. YL: Conceptualization, Funding acquisition, Project administration, Supervision, Writing – review & editing. YC: Funding acquisition, Investigation, Software, Writing – review & editing. XW: Funding acquisition, Methodology, Writing – review & editing.
