# Supplementary material for: Grazing exclusion is more beneficial for restoring soil organic carbon and nutrient balance than afforestation on degraded sandy land
Source: Front Plant Sci. 2023 Dec 21;14:1326244. doi: 10.3389/fpls.2023.1326244 (PMC10764600; doi:10.3389/fpls.2023.1326244)
Supplement: Supplementary file 1 [file DataSheet_1.docx]

**Supplementary file**

**TABLE S1** Characteristics of the sample sites.

| Site type | Elevation (m) | Aspect (°, from north) | Slope (°) | Herbaceous vegetation cover (%) | Silt+clay (%, <0.05 mm) | Soil water content (%) |
| --- | --- | --- | --- | --- | --- | --- |
| SF | 367.8 | 184 | 3 | 85.6 | 33.2 | 9.0 |
| 20GE | 361.2 | 177 | 18 | 61.0 | 9.8 | 2.8 |
| 40GE | 355.5 | 181 | 5 | 74.5 | 27.8 | 4.2 |
| 20CM | 364.9 | 204 | 3 | 53.2 | 23.8 | 5.4 |
| 40CM | 355.0 | 189 | 4 | 63.2 | 16.7 | 2.8 |
| 40PS | 361.3 | 212 | 10 | 35.7 | 16.2 | 5.3 |
| 48PS | 381.1 | 208 | 10 | 74.6 | 17.0 | 1.9 |
| AD | 338.6 | 178 | 11 | 0.4 | 10.7 | 6.1 |

SF, natural sparse-forest grassland; 20GE, 20-year grazing exclusion; 40GE, 40-year grazing exclusion; 20CM, 20-year-old *Caragana microphylla* plantation; 40CM, 40-year-old *Caragana microphylla* plantation; 40PS, 40-year-old *Pinus sylvestris* var. *mongolica* plantation; 48PS, 48-year-old *Pinus sylvestris* var. *mongolica* plantation; AD, active dunes.

**Table S2** Vegetation community characteristics of *Pinus sylvestris var. mongolica* and *Caragana microphylla* plantations.

| Site type | Density (plants m^-2^) | Height (m) | Diameter at breast height (cm) | crown diameter (m) |
| --- | --- | --- | --- | --- |
| 20CM | 0.24 | 2.0 | n/a | 3.0 |
| 40CM | 0.52 | 1.3 | n/a | 1.4 |
| 40PS | 0.1 | 6.1 | 11.8 | n/a |
| 48PS | 1.7 | 7.5 | 12.5 | n/a |

20CM, 20-year-old *Caragana microphylla* plantation; 40CM, 40-year-old *Caragana microphylla* plantation; 40PS, 40-year-old *Pinus sylvestris* var. *mongolica* plantation; 48PS, 48-year-old *Pinus sylvestris* var. *mongolica* plantation. n/a, not applicable.

**TABLE S3** Principal components analysis (PCA) results of topography, vegetation, soil properties, and soil C:N:P stoichiometry in China’s Horqin Sandy Land

| Factors | PC1 | PC2 |
| --- | --- | --- |
| **Topography** |  |  |
| Elevation | 0.80 | −0.12 |
| Slope | 0.72 | 0.42 |
| Aspect | −0.23 | 0.91 |
| Cumulative proportion | 40.0% | 74.3% |
| **Vegetation** |  |  |
| Shannon–Wiener diversity index | 0.77 |  |
| Aboveground living biomass | 0.81 |  |
| Litter biomass | −0.59 |  |
| Cumulative proportion | 53.4% |  |
| **Soil properties** |  |  |
| Silt+clay | 0.73 | −0.01 |
| Soil bulk density | 0.29 | −0.12 |
| Soil water content | 0.74 | 0.11 |
| Field water capacity | 0.65 | 0.59 |
| Saturated water content | 0.13 | 0.91 |
| pH | 0.86 | −0.34 |
| Electrical conductivity | 0.87 | −0.28 |
| Cumulative proportion | 44.5% | 64.4% |
| **C:N:P stoichiometry** |  |  |
| SOC | 0.99 | −0.06 |
| TN | 0.93 | −0.37 |
| TP | 0.80 | −0.49 |
| C:N | 0.51 | 0.82 |
| C:P | 0.89 | 0.43 |
| N:P | 0.94 | 0.00 |
| Cumulative proportion | 73.7% | 94.3% |


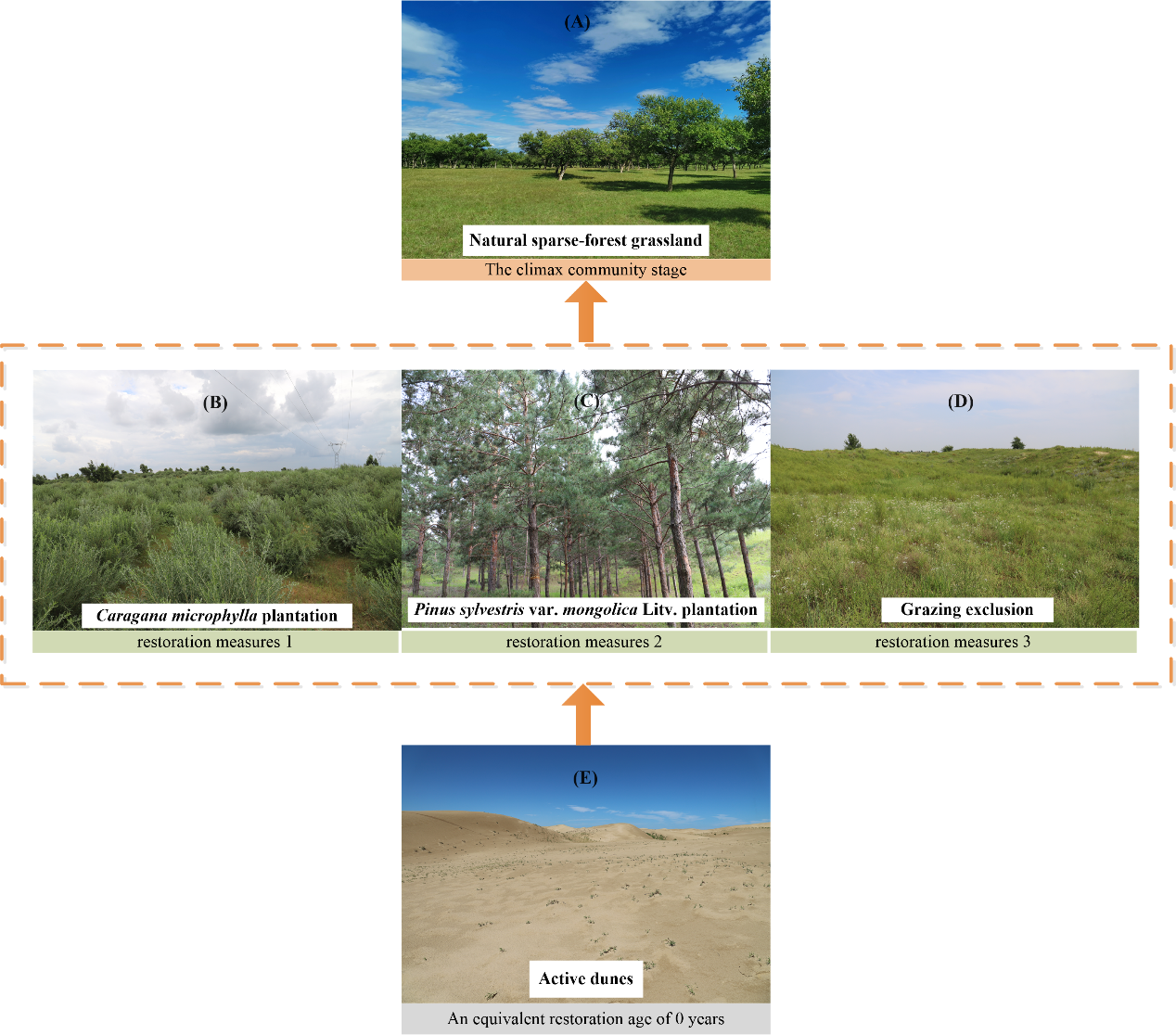


**FIGURE S1** Photographs of selected site types in the Horqin Sandy Land. All images are from the authors’ own collection.

**
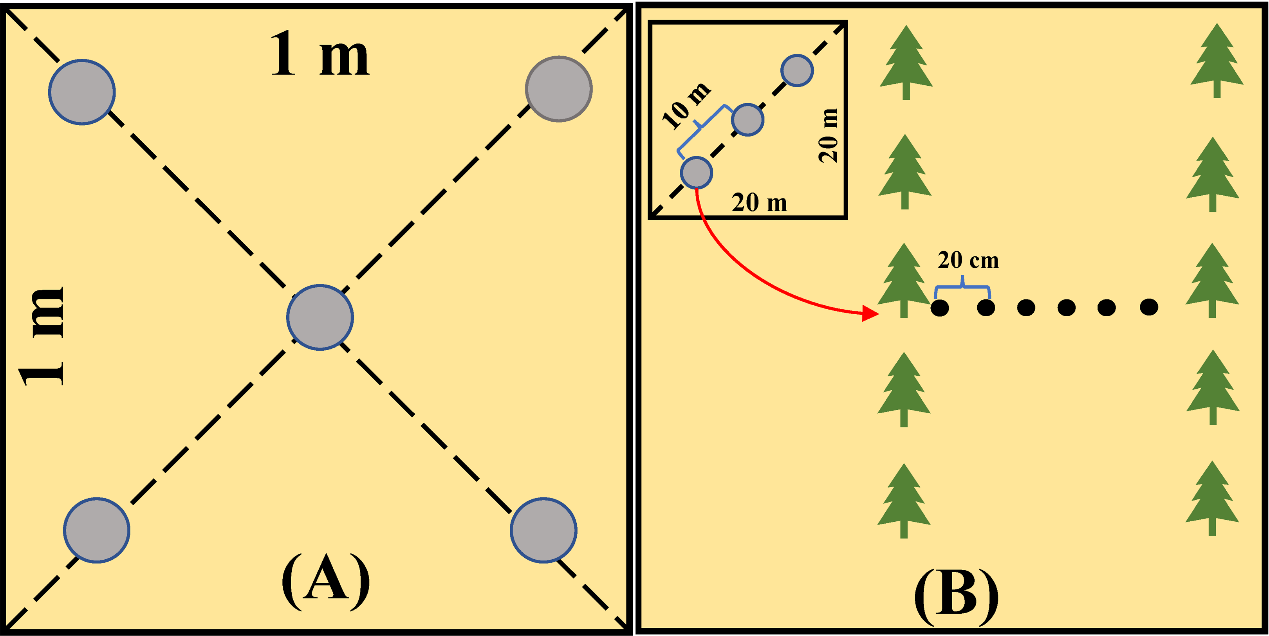
****FIGURE S2** Sampling methods for grazing exclusion, sparse forest, and active dune sites (A), and for *Pinus sylvestris* and *Caragana microphylla* sites (B).


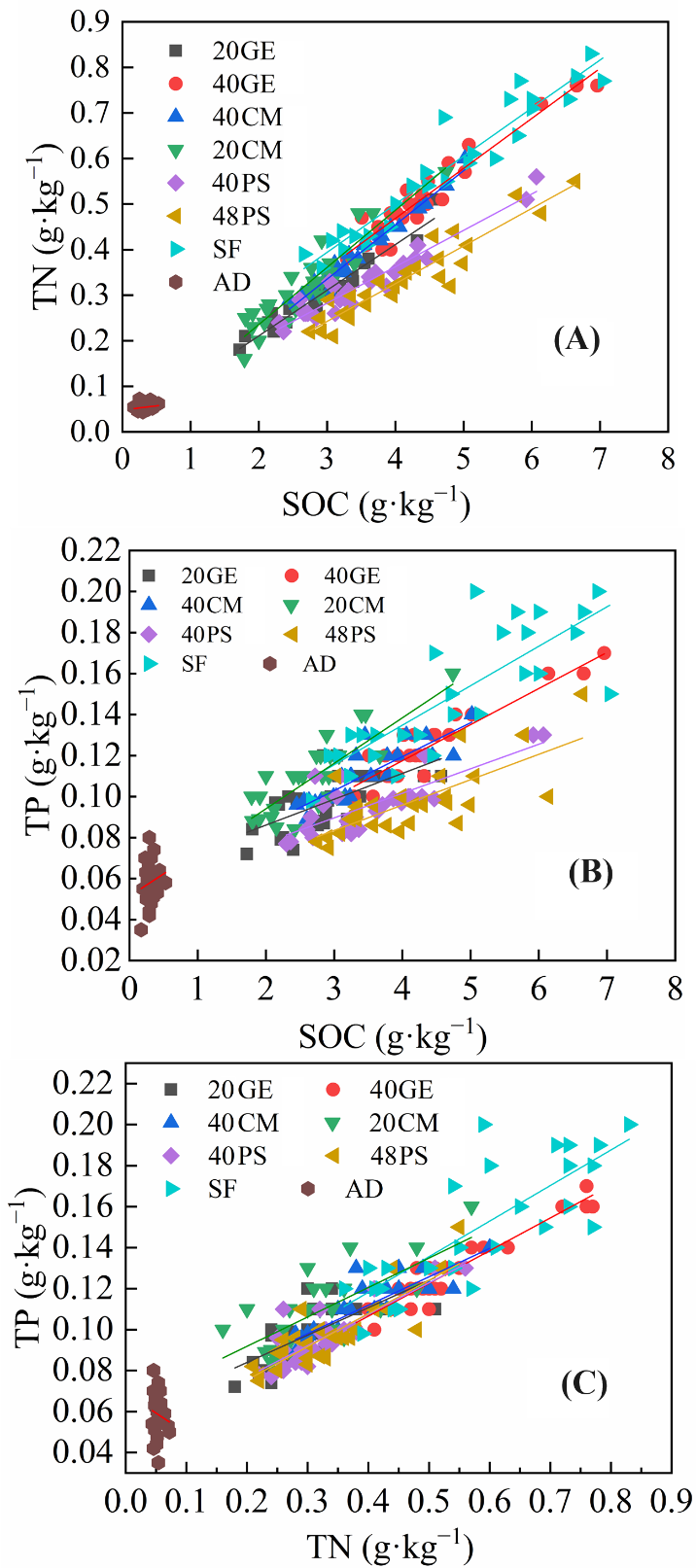


**FIGURE S3** Relationships among soil organic carbon (SOC), total nitrogen (TN), and total phosphorus (TP) for the different site types. 20GE, 20-year grazing exclusion; 40GE, 40-year grazing exclusion; 20CM, 20-year *Caragana microphylla* plantation; 40CM, 40-year *Caragana microphylla* plantation; 40PS, 40-year *Pinus sylvestris* var. *mongolica* plantation; 48PS, 48-year *Pinus sylvestris* var. *mongolica* plantation; SF, natural sparse-forest grassland; AD, active dunes.
